# Association of triglyceride-glucose index with major depressive disorder: A cross-sectional study

**DOI:** 10.1097/MD.0000000000034058

**Published:** 2023-06-16

**Authors:** Man Jin, Peiyuan Lv, Hao Liang, Zhenjie Teng, Chenyang Gao, Xueru Zhang, Aihua Ni, Xiaona Cui, Nan Meng, Litao Li

**Affiliations:** a Department of Neurology, Hebei Medical University, Shijiazhuang, China; b Department of Neurology, Hebei General Hospital, Shijiazhuang, China; c Hebei Provincial Key Laboratory of Cerebral Networks and Cognitive Disorders, Shijiazhuang, China; d Cardiology Department, Hebei General Hospital, Shijiazhuang, China.

**Keywords:** glucose, insulin resistance, major depressive disorder, triglyceride, TyG

## Abstract

The triglyceride-glucose (TyG) index has been proposed as a new marker for insulin resistance, which is associated with a risk of major depressive disorder (MDD). This study aims to explore whether the TyG index is correlated with MDD. In total, 321 patients with MDD and 325 non-MDD patients were included in the study. The presence of MDD was identified by trained clinical psychiatrists using the International Classification of Diseases 10th Revision. The TyG index was calculated as follows: Ln (fasting triglyceride [mg/dL] × fasting glucose [mg/dL]/2). The results revealed that the MDD group presented higher TyG index values than the non-MDD group (8.77 [8.34–9.17] vs 8.62 [8.18–9.01], *P* < .001). We also found significantly higher morbidity of MDD in the highest TyG index group than in the lower TyG index group (59.9% vs 41.4%, *P* < .001). Binary logistic regression revealed that TyG was an independent risk factor for MDD (odds ratio [OR] 1.750, 95% confidence interval: 1.284–2.384, *P* < .001). We further assessed the effect of TyG on depression in sex subgroups. The OR was 3.872 (OR 2.014, 95% confidence interval: 1.282–3.164, *P* = .002) for the subgroup of men. It is suggested that the TyG index could be closely associated with morbidity in MDD patients; thus, it may be a valuable marker for identifying MDD.

## 1. Introduction

Major depressive disorder (MDD) is the most common affective disorder that seriously affects people’s social function and impairs their abilities to function in daily life and the workplace.^[1,2]^ It manifests as low mood, diminished interest, impaired concentration, insomnia, or even intense suicidal ideation for >2 weeks.^[3,4]^ MDD is probably considered a major cause of suicide.^[5]^ A recent study indicated that MDD will become the leading cause of disability-adjusted life years in 2030.^[6,7]^ MDD has been the second leading cause of disability-adjusted life years in China since 2010.^[8]^ Current treatments for depression are symptom-suppressing. According to previous research, only 30% of MDD patients attain complete remission,^[9]^ but 50 to 66% are not able to achieve remission from their first antidepressant treatment.^[10]^ These findings underscore the need for novel treatment interventions.

Peripheral insulin resistance (IR) occurs when peripheral tissues are unable to respond to insulin stimulation, leading to increased peripheral insulin levels.^[11,12]^ Some large observational studies have found that diabetes is associated with depression caused by IR.^[13,14]^ IR might be a metabolic subtype of depression that can be treated with specific drugs.^[15]^ As a reliable surrogate marker of IR, the triglyceride-glucose (TyG) index, which is based on triglycerides and glucose, has been widely used in recent years^[16]^ and has been related to disease prognoses, such as cardiovascular disease and dementia.^[17,18]^ Moreover, altered lipid profiles, increased weight, and elevated blood glucose levels contribute to depressed mood in adults.^[19,20]^ Recently, studies have demonstrated that the TyG index is related to depression progression in China’s elderly and the existence of depressive states in US adults.^[15,20]^ Therefore, we speculated that there might be a certain association between TyG and MDD. In this study, we aimed to evaluate the role of IR in the underlying pathophysiology of depression by assessing the TyG index.

## 2. Materials and methods

### 2.1. Subjects

This study retrospectively analyzed the records of MDD patients and non-MDD patients at the Hebei General Hospital, China. We reviewed information on eligible subjects who were diagnosed with the first episode of MDD and consecutively admitted to our inpatient unit from January 2021 to December 2021 according to records saved in electronic databases. The non-MDD group comprised patients undergoing physical examination during the same period in our hospital. The sociodemographic characteristics of the MDD patients and non-MDD patients were similar. Moreover, the non-MDD patients were never diagnosed with any psychiatric disorders.

The inclusion criteria were as follows: confirmed diagnosis of MDD based on the International Classification of Diseases 10th Revision by trained clinical psychiatrists (only for the MDD group); Han ethnicity, aged between 18 and 65 years; and initial diagnosis of depression.^[21]^

The exclusion criteria were as follows: additional psychiatric diagnoses, such as anxiety disorder, bipolar disorder, alcoholism, and/or drug addiction; unstable or serious medical conditions that may affect fasting glucose or lipids, such as active infection, inflammation, hepatic or renal failure, and diabetes; preexisting diagnosed diabetes or impaired glucose tolerance; and other conditions that may affect fasting glucose and lipid metabolisms, such as pregnancy or breastfeeding.^[4,14]^

**Figure 1.**
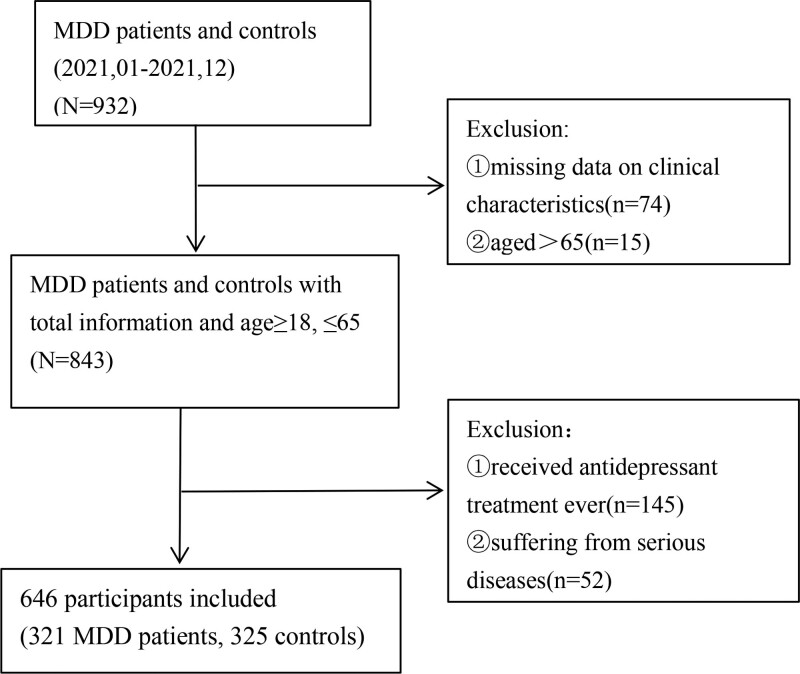
Flowchart of participant selection.

Figure 1 provides further details about the sample, exclusion criteria, and study design. Ultimately, 646 individuals were enrolled. Among them, 321 patients were included in the MDD group, and 325 individuals were included in the non-MDD group. This study followed the principles in the Declaration of Helsinki and was approved by the Ethical Committees of the Hebei General Hospital (NO.2022176).

### 2.2. Data collection and definitions

We extracted the clinical and demographic characteristics, including age, sex, marital status, current smoking, alcohol status, education levels, and family history of psychiatric disorders, hypertension, cancer, stroke, hypothyroidism, and cardiovascular disease, from patients’ electronic medical records or interview records.

Antecubital blood samples for fasting triglycerides and fasting glucose as routine examinations for inpatients were collected after >12 hours of fasting. The formula for calculating the TyG index was TyG = Ln [fasting triglyceride (mg/dL) × fasting glucose (mg/dL)/2].^[14]^

### 2.3. Statistical analysis

All statistical analyses were performed using SPSS version 25.0 (SPSS Inc., Chicago, IL). For normally distributed data, continuous variables are reported as the mean ± standard deviation or median (IQR) for skewed distributed data. One-way analysis of variance or Student *t* test was used to compare normally distributed data, and the Mann–Whitney *U* test was used to compare continuous variables with abnormal distributions. Categorical variables are reported as frequencies and percentages (%) and were compared using the chi-square test (*χ*^2^ test). Binary logistic regression analyses were used to determine the independent risk factors for depression. Binary logistic regression analyses were performed using 3 different models; model 1 did not control any confounders, and model 2 and model 3 were performed to control potential confounders. Odds ratios (ORs) are presented with a 95% confidence interval (CI).

## 3. Results

### 3.1. Patient demographics and clinical characteristics based on MDD

The current study included 646 patients, including 370 females and 276 males. The mean age of the patients was 44 (36,55), ranging from 18 to 65 years. There were 321 patients diagnosed with MDD, and 325 patients were included in the non-MDD group. We observed no differences in sex, body mass index (BMI), marital status, current smoking, alcohol status, education levels, hypertension, cancer, or cardiovascular disease between the 2 groups (all *P* > .05). Compared with subjects without MDD, those with MDD were more likely to be female (*P* < .01) and have a family history of psychiatric disorders (*P* = .001), stroke (*P* = .005) and hypothyroidism (*P* = .028). Mann–Whitney *U* tests showed that the MDD group had higher levels of triglycerides and TyG index values but lower levels of glucose (all *P* < .05). The data are shown in Table 1.

**Table 1:** Patient demographics and clinical characteristics in the major depression disorder and nonmajor depression disorder groups.

Variables	Total (n = 646)	MDD group	Non-MDD group	*P* value
Age, years, median (IQR)	44 (36–55)	43 (21.70–26.17)	47 (35–55)	.209
Sex, female, n (%)	370 (57.3)	209 (65.1)	161 (49.5)	<.001
BMI, mean ± SD	24.15 ± 3.43	24.18 ± 3.47	24.13 ± 3.39	.838
Married, n (%)	531 (82.2)	264 (82.2)	267 (82.2)	.976
Education levels, n (%)				.522
Illiterate	39 (6.0)	15 (4.7)	24 (7.4)	
Primary school	84 (13)	38 (11.8)	46 (14.2)	
Junior high school	159 (24.6)	80 (24.9)	79 (24.3)	
Senior high school	221 (34.2)	114 (35.5)	107 (32.9)	
College or university and above	143 (22.1)	74 (23.1)	69 (21.2)	
Current smoking, n (%)	70 (10.8)	38 (11.8)	32 (11.8)	.415
Alcohol status, n (%)	81 (12.5)	45 (14)	36 (11.1)	.259
Family history of psychiatric disorders, n (%)	77 (11.9)	52 (16.2)	25 (7.7)	.001
Hypertension, n (%)	73 (11.3)	41 (12.8)	32 (9.8)	.240
Cardiovascular disease, n (%)	41 (6.3)	22 (6.9)	19 (5.8)	.600
Cancer, n (%)	40 (6.2)	23 (7.2)	17 (5.2)	.308
Stroke, n (%)	31 (4.8)	23 (7.2)	8 (2.5)	.005
Hypothyroidism, n (%)	27 (4.2)	19 (5.9)	8 (2.5)	.028
Fasting glucose, mg/dL, median (IQR)	92.98 (84.87–101.27)	86.68 (79.56–94.51)	97.49 (91.63–102.62)	<.001
Triglyceride, mg/dL, median (IQR)	127.58 (84.17–190.49)	143.53 (93.03–219.73)	115.18 (73.10–168.78)	<.001
TyG, median (IQR)	8.68 (8.26–9.09)	8.77 (8.34–9.17)	8.62 (8.18–90.1)	<.001

BMI = body mass index, DD = major depression disorder, IQR = interquartile range, MDD = major depression disorder, SD = standard deviation, TyG = triglyceride-glucose.

### 3.2. Baseline demographic and clinical characteristics of patients based on TyG index

We divided participants into 3 subgroups in accordance with the tertiles of the TyG index (Q1 < 8.39, 215 patients; Q2 8.39–8.98, 214 patients; Q3 > 8.98, 217 patients). The participants’ baseline characteristics are shown in Table 2. Indeed, the percentage of MDD patients increased according to the TyG index (*P* < .05), and the numbers of MDD patients were 89 (41.4%), 102 (47.7%), and 130 (59.9%) in Tertile 1, Tertile 2, and Tertile 3, respectively. The proportion of female patients in Tertile 3 was significantly higher than that in Tertile 1 and Tertile 2 (*P* < .001). The average age of patients in Tertile 2 and Tertile 3 was significantly higher than that of patients in Tertile 1. The BMI indexes and education levels of the 3 TyG index levels were significantly different (all *P* < .05).

**Table 2:** Baseline characteristics of patients according to triglyceride-glucose index tertiles.

Variables	Q1 (n = 215)	Q2 (n = 214)	Q3 (n = 217)	*P* value
MDD patients, n (%)	89 (41.4)	102 (47.7)	130 (59.9)	<.001
Age, years, median (IQR)	41 (34–52)	45.5 (37–57)	45 (36.5–55)	.011
Gender, female, n (%)	124 (57.7)	124 (57.9)	165 (76)	<.001
BMI, mean ± SD	23.55 ± 3.46	24.55 ± 3.39	24.36 ± 3.36	.005
Married, n (%)	185 (86)	172 (80.4)	174 (80.2)	.196
Education levels, n (%)				.022
Illiterate or without	20 (9.3)	9 (4.2)	10 (4.6)	
Primary school	24 (11.2)	26 (12.1)	34 (15.7)	
Junior high school	59 (27.4)	44 (20.6)	56 (25.8)	
Senior high school	78 (36.3)	79 (36.9)	64 (29.5)	
College or university and above	34 (15.8)	56 (26.2)	53 (24.4)	
Current smoking, n (%)	20 (9.3)	27 (12.6)	23 (10.6)	.538
Alcohol status, n (%)	25 (11.6)	33 (15.4)	23 (10.6)	.283
Family history of psychiatric disorders, n (%)	21 (9.8)	21 (9.8)	35 (16.1)	.063
Hypertension, n (%)	27 (12.6)	24 (11.2)	22 (10.1)	.729
Cardiovascular disease, n (%)	14 (6.5)	15 (7.0)	12 (5.5)	.814
Cancer, n (%)	17 (7.9)	13 (6.1)	10 (4.6)	.362
Stroke, n (%)	8 (3.7)	12 (5.6)	11 (5.1)	.642
Hypothyroidism, n (%)	11 (5.1)	7 (3.3)	9 (4.1)	.634

BMI = body mass index, IQR = interquartile range, MDD = major depression disorder, SD = standard deviation.

### 3.3. Associations between the TyG index and depression

Binary logistic regression analysis was used to compute the OR between the TyG index and MDD. We used 3 different models to evaluate the ORs of participants with an incidence of MDD. Variables with baseline demographic and clinical characteristics that impact the morbidity of depression were included in the logistic regression. The unadjusted logistic regression analysis (Model 1) showed that the TyG index (OR 1.806; 95% CI: 1.353–2.410, *P* < .001) was positively associated with MDD. After adjustment for age, sex, BMI, marital status, education levels, family history of psychiatric disorders, hypertension, cardiovascular disease, cancer, stroke, and hypothyroidism (Model 3), the TyG index (OR 1.750; 95% CI 1.284–2.384, *P* < .001) was independently associated with the incidence of MDD (Table 3). We further assessed the association of TyG and MDD in sex-based subgroups. After adjustment for confounders, the male subgroup showed a positive association between the TyG index and depression (OR 2.014; 95% CI 1.282–3.164, *P* = .002). However, in the female subgroup, this positive correlation could not be fully established, and the *P* values for the 3 models were 0.051, 0.082, and 0.038, respectively (Table 4).

**Table 3:** Binary logistic regression analysis model of triglyceride-glucose index and major depression disorder.

Variables	Model 1		Model 2		Model 3	
	OR (95% CI)	*P* value	OR (95% CI)	*P* value	OR (95% CI)	*P* value
TyG	1.806 (1.353–2.410)	<.001	1.667 (1.231–2.257)	.001	1.750 (1.284–2.384)	<.001

Model 1: unadjusted.

Model 2: adjusted for age, sex, body mass index (BMI), marital status, family history of psychiatric disorders, and education levels.

Model 3: adjusted for age, sex, BMI, marital status, family history of psychiatric disorders, education levels, hypertension, cardiovascular disease, stroke, cancer, and hypothyroidism.

CI = confidence interval, OR = odds ratio, TyG = triglyceride-glucose index.

**Table 4:** Binary logistic regression analysis model of triglyceride-glucose index and major depression disorder in sex-based subgroups.

Variables	Model 1		Model 2		Model 3	
	OR (95% CI)	*P* value	OR (95% CI)	*P* value	OR (95% CI)	*P* value
Male	2.255 (1.365–3.727)	.002	2.157 (1.316–3.535)	.002	2.014 (1.282–3.164)	.002
Female	1.521 (0.999–2.317)	.051	1.434 (0.956–2.152)	.082	1.507 (1.023–2.219)	.038

Model 1: unadjusted.

Model 2: adjusted for age, sex, body mass index (BMI), marital status, family history of psychiatric disorders, and education levels.

Model 3: adjusted for age, sex, BMI, marital status, family history of psychiatric disorders, education levels, hypertension, cardiovascular disease, stroke, cancer, and hypothyroidism.

CI = confidence interval, OR = odds ratio.

## 4. Discussion

The TyG index was associated with MDD in this cross-sectional study. According to this study, the MDD group showed a high TyG index in comparison with the non-MDD group. When comparing the morbidity of MDD patients with different TyG index levels, we found that the morbidity increased with TyG index levels, and a high TyG index level was associated with higher morbidity of MDD in participants. In addition, our results also revealed that a higher TyG index was a risk factor for depression.

It is still necessary to investigate the mechanism explaining the relationship between the TyG index and MDD in more detail, and several possible and testable mechanisms have been proposed for this association.

The TyG index has been reported to be a predictor of diabetes, hypertension, and metabolic syndrome, signaling clinical metabolic dysregulation and thus a potential mechanism between depression and adverse physical status.^[20]^ Adverse IR is common in depression patients.^[14]^ Peripheral IR can metastasize to the brain, resulting in brain IR,^[10,22]^ which corresponds to a reduced physiological response to insulin in the brain.^[23]^ In neurons, insulin plays pivotal roles in maintaining synaptic function, such as stimulating neurite outgrowth, regulating catecholamine release and uptake, and functioning as a key regulator of n-methyl-d-aspartate and γ-aminobutyric acid receptors.^[24]^ Additionally, insulin promotes neuronal survival and modulates synaptic plasticity by inhibiting apoptosis.^[25]^ In addition to affecting mood and cognition, brain IR can be caused by activating microglia and astrocytes, increasing neuroinflammation, and impairing intracellular insulin signaling in neurons.^[23]^ According to a previous study, the Korean general population showed a correlation between IR and depressive symptoms, especially in young adults and nondiabetic individuals.^[26]^ Another study also showed that depressive symptoms are associated with metabolic derangements, such as the Homeostatic Model Assessment-IR index, an indicator of IR.^[27]^

IR has been measured by using the TyG index as an alternative strategy in recent years.^[14]^ Due to its low cost and accessibility, the TyG index has become widely used.^[28,29]^ Studies have shown that diabetes,^[30]^ hypertension,^[31]^ and nonalcoholic fatty liver disease^[32]^ have been linked to the TyG index, and it may be used to predict cardiovascular events in the future.^[31]^ Furthermore, a study confirmed that the TyG index was independently associated with the presence of mild cognitive impairment in older people.^[33]^ Although the association between depression and IR has been previously studied, there are scant data evaluating this association with the TyG index, particularly among Asians.^[26]^

To our knowledge, Shi et al conducted the first study to examine the correlation between TyG and depression in the general population. Their study involved >13,000 people in the US and indicated that depressive symptoms were significantly associated with a higher TyG index, and the OR increased with the TyG index.^[14]^ We demonstrated for the first time that the TyG index has a positive association with the morbidity of MDD in the Asian population. Similarly, we also found higher morbidity of MDD patients at the highest level of the TyG index. The TyG index could be closely related to the pathogenesis of MDD.

According to our results, the MDD group had a higher level of triglycerides. Some studies have shown that triglyceride levels are closely linked to depressive disorder.^[34]^ A meta-analysis involving 11 case–control studies indicated that compared to healthy controls, patients with first-episode MDD were significantly associated with higher triglycerides.^[35]^ As a result of increased sensitivity to both environmental and psychological stress, MDD is frequently associated with hypercortisolemia. Most importantly, it has emerged as a major pathophysiological link between major depression and metabolic syndrome.^[36]^ In those with chronic major depression, where hypercortisolemia occurs chronically, the body may provide the energy for the “fight or flight” response for a short time by promoting glucocorticoid secretion, which can have a major impact on the metabolism of carbohydrates, lipids, and proteins. Adipose tissue mobilizes lipids and deposits them in the abdominal adipose tissue when this occurs.^[37]^

Indeed, improvement of insulin sensitivity has been postulated as a novel antidepressant mechanism.^[38,39]^ A study demonstrated that metformin, which belongs to a class of insulin-sensitizing drugs, may act by decreasing circulating branched-chain amino acid levels to improve peripheral IR and favor serotonergic neurotransmission in the hippocampus, consequently promoting antidepressant-like effects in mice.^[19,39]^ Another study revealed that after 12 weeks of rosiglitazone treatment, 12 patients with depressive disorder exhibited significant declines in both depression severity and the IR index. These results implied the vital role of IR in the pathogenesis of depression and its potential novel use as an insulin-sensitizing agent in the treatment of depressive disorders.^[19]^

The binary logistic regression analysis stratified by sex subgroups in our study was particularly interesting. Within the female subgroup, depression did not appear to be significantly associated with TyG. We did not fully understand the underlying etiology of this finding, but it suggested that there is a strong positive correlation between TyG and MDD in nondiabetic male individuals.

It is also important to address several limitations of this study. First, because of the cross-sectional design, we cannot speculate on the causal relationship directly, which requires verification by a prospective cohort study. Second, the patients included in the current study were from a Han Chinese population and were limited to hospitalized patients with MDD. Consequently, our findings should be replicated in a population of other ethnically and clinically diverse backgrounds. Third, the number of samples was relatively small due to the complexity and limited information on the course of the disease. Despite possible selection bias, the results were still consistent after adjusting for different variables. Despite some flaws in the sample selection, our conclusions remained valid. Future multicenter cohort studies on MDD and TyG are warranted to validate the results of this study.

## 5. Conclusion

According to our study, the TyG index was notably associated with depression, especially in men. This result suggested that primary interference with metabolic health might also promote mental and spiritual health. The TyG index may be a new index of depression progress, providing new perspectives for both primary preventive and interventive mental wellness. A better understanding of the mechanisms underlying the TyG index and MDD interaction will enable drug development aimed at treating and/or preventing severe disorders.

## Author contributions

**Conceptualization:** Litao Li.

Data curation: Man Jin, Hao Liang, Chenyang Gao, Xueru Zhang.

Formal analysis: Man Jin, Hao Liang, Zhenjie Teng.

Funding acquisition: Peiyuan Lv, Litao Li.

Investigation: Man Jin, Aihua Ni, Xiaona Cui, Nan Meng.

Methodology: Man Jin, Zhenjie Teng.

Supervision: Peiyuan Lv, Litao Li.

Writing – original draft: Man Jin.

Writing – review & editing: Man Jin, Peiyuan Lv, Litao Li.
